# P-1612. Does the Use of Diagnostic Testing Vary by Race and Social Vulnerability for those Seen in the Emergency Department or Admitted to the Hospital? A Retrospective Analysis in a Large Healthcare System

**DOI:** 10.1093/ofid/ofae631.1779

**Published:** 2025-01-29

**Authors:** Maria Mupanomunda, Reese Cosimi, Stacy D Garrett-Ray, Florian Daragjati, Subhangi Ghosh, Frederick Masoudi, Allison Bollinger, Thomas Aloia, Richard Fogel, Mohamad G Fakih

**Affiliations:** Ascension, Gambrills, Maryland; Ascension, Gambrills, Maryland; Ascension, Gambrills, Maryland; Ascension, Gambrills, Maryland; Ascension, Gambrills, Maryland; Ascension/ Chief Science Officer, VP of Research and Anlaytics, St. Louis, Missouri; Ascension, Gambrills, Maryland; Ascension, Gambrills, Maryland; Ascension, Gambrills, Maryland; Ascension, Gambrills, Maryland

## Abstract

**Background:**

Diagnostic stewardship complements antimicrobial stewardship by promoting appropriate use of diagnostic tools. While disparities have been reported in antibiotic prescribing and hospital-acquired infections, data are limited on diagnostic test utilization. We evaluated the association of race and social vulnerability index (SVI) on ordering of diagnostic testing in a large diverse health system.

**Table 1:**
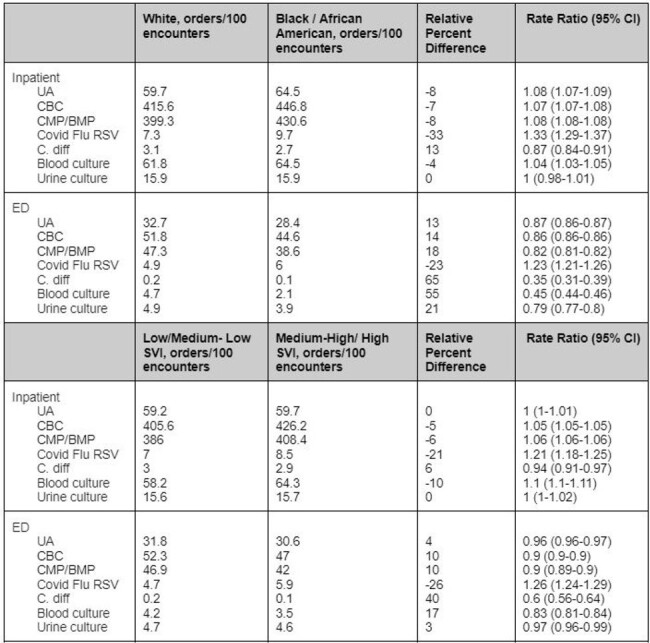
Diagnostic Test Utilization by Race and SVI Inpatient and Observation encounters

**Methods:**

Retrospective analysis of tests ordered for all Inpatient and Emergency Department (ED) encounters of 85 hospitals in a large health system between January 1, 2023 to December 31, 2023. The primary clinical outcome was diagnostic test utilization defined as the number of tests ordered per 100 encounters for the following: blood cultures, urinalysis (UA), urine cultures, *Clostridioides difficile* (C. diff), complete blood count (CBC), comprehensive and basic metabolic panels (CMP/BMP), and COVID-Influenza-RSV panel. Data were stratified across patient race and SVI.

**Results:**

A total of 751,638 Inpatient (70% White, 18% Black/African American (AA)) and 2,440,769 ED (60% White, 27% Black/AA) encounters were included in the analysis. Inpatient White patients had higher rates of diagnostic testing for C. diff than Black/AA patients (relative difference, 16%; p< 0.001) while UA, CBC, CMP/BMP, blood culture, and COVID-Influenza-RSV panel ordering was lower in White patients (relative difference, -6, -5, -5, -38; p< 0.001, respectively). In the ED, test ordering was higher in White patients in all test panels except for COVID Flu RSV. Similar differences seen by race were observed by SVI, with Low/Medium-Low SVI mirroring testing in White patients and High/Medium-High SVI mirroring testing in Black/AA patients.

**Conclusion:**

Differences were identified in diagnostic test utilization by race and SVI in the inpatient and ED settings. Additional factors such as age, diagnosis, and regional variability may need to be further examined in future analyses. Although these disparities were observed, our internal mortality data shows no differences by race. Further investigation is warranted to determine appropriateness of diagnostic test ordering in each population to better interpret the disparities identified in this study.

**Disclosures:**

**All Authors**: No reported disclosures

